# Trends in US Surgical Procedures and Health Care System Response to Policies Curtailing Elective Surgical Operations During the COVID-19 Pandemic

**DOI:** 10.1001/jamanetworkopen.2021.38038

**Published:** 2021-12-08

**Authors:** Aviva S. Mattingly, Liam Rose, Hyrum S. Eddington, Amber W. Trickey, Mark R. Cullen, Arden M. Morris, Sherry M. Wren

**Affiliations:** 1 Stanford University School of Medicine, Stanford, California; 2 Health Economics Resource Center, Department of Veterans Affairs, Palo Alto, California; 3 Stanford-Surgery Policy Improvement Research and Education Center, Stanford, California; 4 Stanford Center for Population Health Sciences, Stanford, California; 5 Surgical Service, Palo Alto Veterans Affairs Health Care System, Palo Alto, California; 6 Department of Surgery, Stanford University School of Medicine, Stanford, California

## Abstract

**Importance:**

The COVID-19 pandemic has affected every aspect of medical care, including surgical treatment. It is critical to understand the association of government policies and infection burden with surgical access across the United States.

**Objective:**

To describe the change in surgical procedure volume in the US after the government-suggested shutdown and subsequent peak surge in volume of patients with COVID-19.

**Design, Setting, and Participants:**

This retrospective cohort study was conducted using administrative claims from a nationwide health care technology clearinghouse. Claims from pediatric and adult patients undergoing surgical procedures in 49 US states within the Change Healthcare network of health care institutions were used. Surgical procedure volume during the 2020 initial COVID-19–related shutdown and subsequent fall and winter infection surge were compared with volume in 2019. Data were analyzed from November 2020 through July 2021.

**Exposures:**

2020 policies to curtail elective surgical procedures and the incidence rate of patients with COVID-19.

**Main Outcomes and Measures:**

Incidence rate ratios (IRRs) were estimated from a Poisson regression comparing total procedure counts during the initial shutdown (March 15 to May 2, 2020) and subsequent COVID-19 surge (October 22, 2020-January 31, 2021) with corresponding 2019 dates. Surgical procedures were analyzed by 11 major procedure categories, 25 subcategories, and 12 exemplar operative procedures along a spectrum of elective to emergency indications.

**Results:**

A total of 13 108 567 surgical procedures were identified from January 1, 2019, through January 30, 2021, based on 3498 Current Procedural Terminology (CPT) codes. This included 6 651 921 procedures in 2019 (3 516 569 procedures among women [52.9%]; 613 192 procedures among children [9.2%]; and 1 987 397 procedures among patients aged ≥65 years [29.9%]) and 5 973 573 procedures in 2020 (3 156 240 procedures among women [52.8%]; 482 637 procedures among children [8.1%]; and 1 806 074 procedures among patients aged ≥65 years [30.2%]). The total number of procedures during the initial shutdown period and its corresponding period in 2019 (ie, epidemiological weeks 12-18) decreased from 905 444 procedures in 2019 to 458 469 procedures in 2020, for an IRR of 0.52 (95% CI, 0.44 to 0.60; *P* < .001) with a decrease of 48.0%. There was a decrease in surgical procedure volume across all major categories compared with corresponding weeks in 2019. During the initial shutdown, otolaryngology (ENT) procedures (IRR, 0.30; 95% CI, 0.13 to 0.46; *P* < .001) and cataract procedures (IRR, 0.11; 95% CI, −0.11 to 0.32; *P* = .03) decreased the most among major categories. Organ transplants and cesarean deliveries did not differ from the 2019 baseline. After the initial shutdown, during the ensuing COVID-19 surge, surgical procedure volumes rebounded to 2019 levels (IRR, 0.97; 95% CI, 0.95 to 1.00; *P* = .10) except for ENT procedures (IRR, 0.70; 95% CI, 0.65 to 0.75; *P* < .001). There was a correlation between state volumes of patients with COVID-19 and surgical procedure volume during the initial shutdown (*r* = −0.00025; 95% CI, −0.0042 to −0.0009; *P* = .003), but there was no correlation during the COVID-19 surge (r = −0.00034; 95% CI, −0.0075 to 0.00007; *P* = .11).

**Conclusions and Relevance:**

This study found that the initial shutdown period in March through April 2020, was associated with a decrease in surgical procedure volume to nearly half of baseline rates. After the reopening, the rate of surgical procedures rebounded to 2019 levels, and this trend was maintained throughout the peak burden of patients with COVID-19 in fall and winter; these findings suggest that after initial adaptation, health systems appeared to be able to self-regulate and function at prepandemic capacity.

## Key Points

**Question:**

Were 2 separate COVID-19 crises, one policy driven during the initial shutdown and the other occurring during the highest burden of infections, associated with changes in surgical procedure volume in the US surgical health system?

**Findings:**

In this cohort study of more than 13 million US surgical procedures from January 1, 2019, through January 30, 2021, there was a 48.0% decrease in total surgical procedure volume immediately after the March 2020 recommendation to cancel elective surgical procedures. Surgical volume returned to 2019 rates in all surgical specialties except otolaryngology, a rate maintained during the COVID-19 peak surge in fall and winter.

**Meaning:**

These findings suggest that health systems learned to adapt and were able to self-regulate, maintaining surgical procedure volume during the largest peak in volume of patients with COVID-19.

## Teaser

This cohort study compares surgical procedure volume after the COVID-19–related shutdown of elective procedures and during the period of peak COVID-19 infection rates with prepandemic procedure volume.

## Introduction

In February 2020, US physicians and public health personnel watched in real time the mounting deaths among patients and health care workers with COVID-19 and the associated resource shortages in Europe.^1,2^ Soon thereafter, the New York City metropolitan area became the first US epicenter for COVID-19. The most recent pandemic the US had faced, the 2009 influenza A (H1N1) virus pandemic was associated with mortality (0.02%) and hospitalization (0.45%) rates of less than one-half of 1 percent of the estimated 60.8 million people infected.^3^ In contrast, COVID-19 was associated with unprecedented stress and demands on the New York City health system, with increased rates of mortality (9.6%) and hospitalization (26.6%).^4^ On March 13, 2020, the US president declared a national emergency, leading to a shutdown of all nonessential activities throughout the United States.^5^ The American College of Surgeons (ACS) and other major surgical specialty societies recommended minimizing, postponing, or canceling elective surgical procedures in mid-March and published guidelines for triage of elective procedures by surgical specialty.^6,7^ The Centers for Medicare & Medicaid Services (CMS) and US Surgeon General also issued statements and recommendations for postponement of nonessential surgical procedures.^6,8^ Recommendations were driven by concerns that continuation of elective surgical treatments could potentially compromise hospital and intensive care unit (ICU) capacity and result in shortages in personal protective equipment (PPE) supplies. In line with national recommendations, 35 states had formal declarations by state governors or medical societies to postpone all nonessential surgical procedures, which was associated with a decrease in surgical procedure volume during the initial months of the pandemic shutdown.^9^

The US had no framework, systems, or processes for a sudden contraction in surgical procedure volume. Nonetheless, 35 days after the ACS recommendation to curtail elective procedures, a new joint statement was published from the ACS, American Society of Anesthesiologists, Association of periOperative Registered Nurses, and American Hospital Association providing guidance for resumption of elective surgical procedures.^10^ CMS similarly released the “Opening Up America Again” guideline.^11^ Hospitals developed processes to reopen elective surgical procedure access; for example, in Veterans Affairs hospitals, surgical procedures across all specialties rebounded in May through June 2020, albeit not to levels of the previous year.^12^ During subsequent months, as the volume of patients with COVID-19 surged higher in the so-called second wave, regulation of surgical procedure scheduling was left to states and individual hospital systems

The purpose of this study was to examine the association of 2 distinct COVID-19–related crises, one policy driven during the initial shutdown and the other related to the statewide burden of infections at each period, with surgical procedure volume in US surgical system. We used a large, nationwide claims data set to compare surgical procedure volume and rates during the 2020 government-led initial shutdown and subsequent fall and winter COVID-19 surge with the same periods during 2019.

## Methods

This retrospective cohort study used claims data from a nationwide health care technology clearinghouse to examine rates, frequency, and types of surgical procedures performed during the 2020 COVID-19 pandemic compared with claims in 2019, a nonpandemic year. This study was approved by the Stanford University Institutional Review Board, and a waiver of informed consent was granted because the data were deidentified. This study followed Strengthening the Reporting of Observational Studies in Epidemiology (STROBE) reporting guideline for cohort studies.

### Data Sources

Deidentified claims were provided by Change Healthcare, a US health care technology company, for use limited to COVID-19 research. This data set is part of the COVID-19 Research Database consortium, a cross-industry collaborative of deidentified data provided pro bono to facilitate COVID-19 research.^13^Data are deidentified and certified by expert determination in accordance with the US Health Insurance Portability and Accountability Act (HIPAA). No identifying information of individuals or covered health care institutions were provided. Commercial claims are available in the data set within 1 day of claim processing and are updated as they are adjudicated.

This study included claims filed from January 1, 2019, to January 30, 2021, in order to capture 12 months of baseline data in 2019 (ie, prepandemic data) and data through January 30, 2021, during the peak COVID-19 burden in the US. Data were included from all states, except Vermont, owing to a significant change in hospitals participating with Change Healthcare between study years.

In addition to claims data, we obtained publicly available 7-day cumulative incidence rates of individuals with COVID-19 per 100 000 members of the population from the Centers for Disease Control and Prevention COVID Data Tracker.^14^ State data from up to January 30, 2021, were included.

### Cohort

Participants included all individuals who had a claim filed for a surgical procedure during the specified period. We identified all incident professional claims with at least 1 Current Procedural Terminology (CPT) level I surgical code, as defined in a subsequent section. Professional claims without any surgical procedures were excluded. For duplicate claims, the claim with the most recent received date was used. Additionally, only the first surgical claim per patient per calendar day was included to avoid double counting different claims associated with the same surgical event.

### Measures

The primary outcome was the rate of surgical procedures. A surgical procedure was defined as a procedure that would be expected to be performed in an operating room and that included an incision, based on expert discretion. Level I surgical CPT codes from 10030 to 69979 were evaluated by the study team for inclusion. The following procedures were excluded: injections, biopsies, fine-needle aspiration, closed treatments without skin incision (eg, closed treatment of fracture), percutaneous procedures, gastroscopy, colonoscopy, bronchoscopy, and catheter insertions. Ophthalmology procedures were excluded, except for cataract surgical procedures. Those procedures not requiring an operating room were excluded from our analysis, as were operations that were classified as non-OR procedures per the Healthcare Cost and Utilization Project (HCUP) Clinical Classifications Software for Services and Procedures version 2020.1 (HCUP).^15^ CPT codes for other and unlisted procedures without further details were excluded.

### Surgical Procedure Categories

#### Major Categories and Subcategories

We defined 11 major surgical procedure categories and 25 subcategories of CPT codes, guided by the HCUP Clinical Classification system. Multiple HCUP clinical areas were combined to create major categories, defined as cardiovascular; cataract; ear, nose, and throat (ENT); general surgical; musculoskeletal; nervous system; obstetrics and gynecology; skin; thoracic; transplant; and urology procedures. We also performed an analysis to evaluate specific procedures within major categories; these specific procedures are referred to as subcategories.

#### Exemplar Procedures

We performed a focused analysis on 12 exemplar procedures. These high-volume procedures were selected to be representative of surgical procedures that range from always elective to mixed elective and urgent to always urgent or emergent. Cataract repair, bariatric surgical treatment, knee arthroplasty, and hip arthroplasty represented always elective procedures; laminectomy, spinal fusion, coronary artery bypass graft, groin hernia repair, and thyroidectomy represented mixed elective and urgent procedures; appendectomy, cesarean delivery, and lower extremity amputation represented always urgent or emergent procedures.

### Statistical Analysis

We compared procedure rates by major category, subcategory, and 12 procedures of interest during 2 key periods, defined as initial shutdown (epidemiological calendar weeks 12-18, 2020; March 15-May 2, 2020) and subsequent COVID-19 surge (week 44, 2020, to week 4, 2021; October 25, 2020-January 30, 2021). The initial shutdown period was selected to encompass the period in which most states had governor directives to postpone elective surgical procedures and for which there were previously published data from the Veterans Health Administration.^9,12^ We estimated incidence rate ratios (IRRs) with 95% CIs from Poisson regression by comparing total procedure counts during these periods with the corresponding weeks in 2019. All regression models included week-of-year fixed effects, and standard errors were clustered at the week level. Percentage changes in volume when reported in the text are derived from the IRRs rather than the using the absolute number of procedures.

We analyzed surgical IRR as a function of COVID-19 infection burden. COVID-19 burden was calculated as mean 7-day cumulative incidence rate per 100 000 population members during the specified period (ie, initial shutdown or COVID-19 surge) for each state. We calculated IRR for each state in both periods. We then separately estimated the linear correlation between the per capita incidence of individuals with COVID-19 and state-specific IRR in each period.

Statistical significance was assessed at the level of *P* < .05, and *P* values were 2-sided. Statistical analysis was performed using R statistical software version 4.0.3 (R Project for Statistical Computing). Data were analyzed from November 2020 through July 2021.

## Results

The study cohort included individuals who underwent 13 108 567 surgical procedures: 6 651 921 surgical procedures in 2019; 5 973 573 surgical procedures in 2020; and 483 073 surgical procedures in January 2021 based on 3498 CPT codes. Comparing full calendar year 2019 with 2020, there were 3 516 569 procedures among women [52.9%] vs 3 156 240 procedures among women [52.8%], with similar age distributions for procedures among pediatric patients (613 192 procedures [9.2%] vs 482 637 procedures [8.1%]) and among patients aged 65 years and older (1 987 397 procedures [29.9%] vs 1 806 074 procedures [30.2%]). There was a similar representation across all US census regions (Table 1). There were 678 348 fewer procedures in 2020 than in 2019, representing a 10.2% reduction for calendar year 2020.

**Table 1.** Population Characteristics

Characteristic	Patients, No. (%)		Change in 2020 vs 2019, %^b^
	January 1-December 31, 2019	January 1-December 31, 2020^a^	
Total patients undergoing surgical treatment^c^	6 651 921 (100)	5 973 573 (100)	−10.2
Sex of patient			
Women	3 516 569 (52.9)	3 156 240 (52.8)	−10.2
Men	3 133 462 (47.1)	2 815 598 (47.1)	−10.1
Missing or unknown	1890 (0.03)	1735 (0.03)	−8.2
Age of patient, y			
<18	613 192 (9.2)	482 637 (8.1)	−21.3
18-49	2 131 729 (32.0)	1 943 830 (32.5)	−8.8
50-64	1 816 497 (27.3)	1 647 729 (27.6)	−9.3
65-79	1 554 545 (23.4)	1 421 468 (23.8)	−8.6
≥80	432 852 (6.5)	384 606 (6.4)	−11.1
Missing or unknown	103 106 (1.6)	93 303 (1.6)	−9.5
Census region			
Northeast	1 411 226 (21.2)	1 157 462 (19.4)	−18.0
Midwest	1 816 038 (27.3)	1 632 979 (27.3)	−10.1
West	1 051 524 (15.8)	995 559 (16.7)	−5.3
South	2 373 133 (35.7)	2 187 573 (36.6)	−7.8

^a^ While data in this study extended to January 30, 2021, this table depicts only calendar year 2019 and 2020 in order to make yearly comparisons of absolute values.

^b^ Difference in absolute volume as a percentage of 2019 volume.

^c^ Represents claims filed; only the first patient claim per calendar day was included. Vermont was excluded, as explained in Methods.

### Surgical Procedure Volume and Category Mix

**Figure 1.**
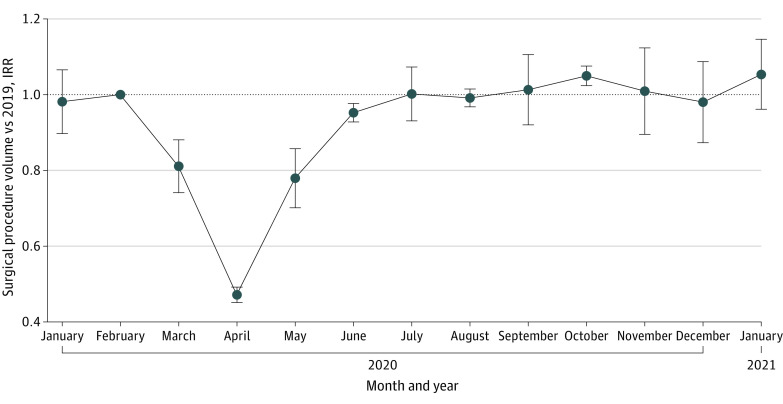
Surgical Procedure Volume Over Time as a Proportion of 2019 Volume Incidence rate ratios (IRRs) and 95% CIs (error bars) were estimated from Poisson regression by comparing total procedure counts during epidemiological weeks with corresponding weeks in 2019. All regression models included week-of-year fixed effects, and standard errors were clustered at the week level. Surgical procedure volume across all categories combined showed a significant decrease in 2020 compared with 2019 in March through June, as represented by IRR over time on the graph. IRR was not significantly different than 1.0 from July through January, indicating no change from 2019 procedure volume.

**Figure 2.**
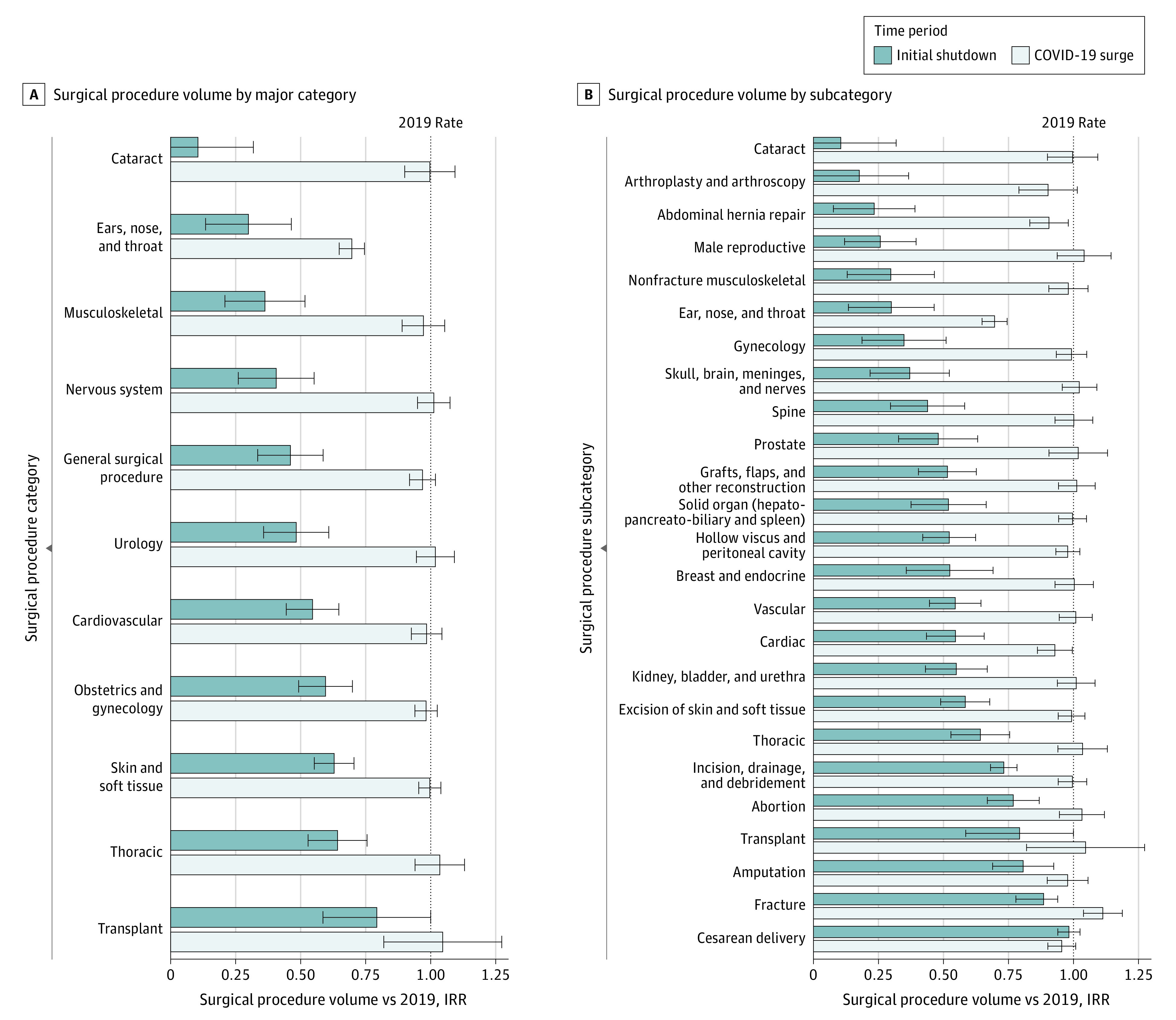
Surgical Procedure Volume During Initial Shutdown and COVID-19 Surge Compared With Prepandemic Rates Initial shutdown indicates March 15 through May 2, 2020; COVID-19 surge, October 25, 2020, through January 30, 2021; IRR, incidence rate ratio showing change in procedure volume from 2019 to 2020, estimated from Poisson regression by comparing total procedure counts during epidemiological weeks in 2020 with corresponding weeks in 2019; error bars, 95% CIs. A, During the initial shutdown period, all major surgical procedure categories except transplant had a significant decrease in volume compared with 2019. During the COVID-19 surge, all major surgical procedure categories, except ears, nose, and throat, were not different from 2019 procedure rates. See eTable 1 in the Supplement for exact values. B, Dark bars indicate change in volume from 2019 during the initial shutdown, which was significantly decreased for all subcategories except transplant and cesarean delivery; light bars, change in procedure volume from 2019 during the COVID-19 surge in fall and winter, which was not different between years except for procedures classified as ears, nose, and throat and abdominal hernia repair. See eTable 2 in the Supplement for exact values.

The overall rate of procedures during the 2020 initial shutdown decreased by 48.0% compared with its corresponding period in 2019 (905 444 procedures in 2019 vs 458 469 procedures in 2020; IRR, 0.52; 95% CI, 0.44 to 0.60; *P* < .001) (Figure 1; eTable 1 in the Supplement). There was a decrease in surgical procedure volume across all major surgical procedure categories compared with the same epidemiological weeks in 2019 (Figure 2A; eTable 1 in the Supplement). Among 11 major surgical procedure categories, the greatest decreases from 2019 to 2020 were in cataract (13 564 procedures vs 1396 procedures; IRR, 0.11; 95% CI, −0.11 to 0.32; *P* = .03), ENT (36 702 procedures vs 10 945 procedures; IRR, 0.30; 95% CI, 0.13 to 0.46; *P* < .001), and musculoskeletal procedures (150 145 procedures vs 53 473 procedures; IRR, 0.36; 95% CI, 0.21 to 0.52; *P* < .001), for overall decreases of 89.5%, 70.1%, and 63.7%, respectively, in 2020 (eTable 1 in the Supplement). The smallest decrease in surgical procedure volume during the initial shutdown was among transplant surgical procedures, with a 20.7% decrease (544 procedures vs 398 procedures; IRR, 0.79; 95% CI, 0.59 to 1.00; *P* = .08), which was not a statistically significant change. Analysis of 25 surgical subcategories found more specific trends within the major surgical procedure categories (Figure 2B; eTable 2 in the Supplement): Cataract surgical procedures, with a decrease of 89.5% (13 564 procedures vs 1396 procedures; IRR, 0.11; 95% CI, −0.11 to 0.32; *P* = .03), and joint arthroplasty, with a decrease of 82.1% (53 328 procedures vs 9737 procedures; IRR, 0.18; 95% CI, −0.01 to 0.37; *P* = .001), had the largest decreases during the initial shutdown period. In contrast, from 2019 to 2020, the rate of cesarean delivery procedures did not change (32 345 procedures vs 30 398 procedures; IRR, 0.98; 95% CI, 0.94 to 1.03; *P* = .42) and the rate of surgical procedures for bone fractures decreased by 14.1% (25 429 procedures vs 19 887 procedures; IRR, 0.86; 95% CI, 0.78 to 0.94; *P* = .001).

During the COVID-19 surge, the overall rate of surgical procedures rebounded to 2019 baseline rates (797 510 procedures vs 756 377; IRR, 0.97; 95% CI, 0.95 to 1.00; *P* = .10) (Figure 1; eTable 1 in the Supplement). This pattern was observed across all major surgical procedure categories and subcategories except for ENT, which had a persistent decrease of 30.3% (60 090 procedures in 2019 vs 41 701 procedures during the surge; IRR, 0.70; 95% CI, 0.65-0.75; *P* < .001) and abdominal hernia repair, which had a persistent 9.4% decrease (52 330 procedures vs 46 484 procedures ; IRR 0.91; 95% CI, 0.83-0.98; *P* = .02) (Figure 2 A and B). In some subcategories, the rate of surgical procedures surpassed 2019 rates; for example, fracture surgical procedure volume increased by 11.3% during the surge (47 585 procedures vs 48 215 procedures; IRR, 1.11; 95% CI 1.04-1.19; *P* = .002) (eTable 2 in the Supplement).

### Exemplar Procedures

During the initial shutdown, 4 procedures with the largest rate decreases vs 2019 were cataract repair (13 564 procedures vs 1396 procedures; IRR, 0.11; 95% CI, −0.11 to 0.32; *P* = .03), bariatric surgical procedures (5697 procedures vs 630 procedures; IRR, 0.12; 95% CI, −0.06 to 0.30; *P* = .006), knee arthroplasty (20 131 procedures vs 2667 procedures; IRR, 0.13; 95% CI, −0.07 to 0.32; *P* = .009), and hip arthroplasty (12 578 procedures vs 2525 procedures; IRR, 0.19; 95% CI, 0.01 to 0.37; *P* < .001) (Table 2; eFigure in the Supplement). A decrease was observed in groin hernia repairs (12 378 procedures vs 2815 procedures; IRR, 0.23; 95% CI, 0.05 to 0.41; *P* < .001), thyroidectomy (2652 procedures vs 985 procedures; IRR, 0.38; 95% CI, 0.22 to 0.55; *P* < .001), spinal fusion (3859 procedures vs 1592 procedures; IRR, 0.42; 95% CI, 0.25 to 0.59; *P* < .001), laminectomy (3199 procedures vs 1512 procedures; IRR, 0.51; 95% CI, 0.34 to 0.68; *P* < .001), and coronary artery bypass graft (3099 procedures vs 1624 procedures; IRR, 0.61; 95% CI, 0.45 to 0.76; *P* < .001). Appendectomy was among the procedures most preserved during the shutdown but still demonstrated a statistically significant 28.8% decrease in volume (10 581 procedures vs 7304 procedures; IRR, 0.71; 95% CI, 0.64 to 0.78; *P* < .001), while lower extremity amputation and cesarean delivery showed no statistically significant change from baseline. In contrast, during the COVID-19 surge, no procedures showed a statistically significant change from the 2019 baseline, except for a 14.3% decrease for knee arthroplasty procedures (40 637 procedures to 36 619 procedures; IRR, 0.86; 95% CI, 0.73 to 0.98; *P* = .04) and an 7.8% decrease for groin hernia repairs (23 625 procedures vs 21 391 procedures; IRR, 0.92; 95% CI, 0.86 to 0.99; *P* = .03) (Table 2; eFigure in the Supplement).

**Table 2.** Volume and IRR of Exemplar Procedures During Initial Shutdown and COVID-19 Surge Compared With Prepandemic Rates

Surgical procedure	Initial shutdown^a^				COVID-19 surge^b^			
	Procedure volume, No.		IRR (95% CI)	*P* value	Procedure volume, No.		IRR (95% CI)	*P* value
	2019	2020			2019	2020		
Cataract repair	13 564	1396	0.11 (−0.11 to 0.32)	.03	24 430	23 797	1.00 (0.90 to 1.09)	.95
Bariatric surgical procedure	5697	630	0.12 (−0.06 to 0.30)	.006	11 148	9371	0.89 (0.75 to 1.03)	.15
Knee arthroplasty	20 131	2667	0.13 (−0.07 to 0.32)	.009	40 637	36 619	0.86 (0.73 to 0.98)	.04
Hip arthroplasty	12 578	2525	0.19 (0.01 to 0.37)	<.001	24 356	22 961	0.90 (0.77 to 1.04)	.18
Groin hernia repair	12 378	2815	0.23 (0.05 to 0.41)	<.001	23 625	21 391	0.92 (0.86 to 0.99)	.03
Thyroidectomy	2652	985	0.38 (0.22 to 0.55)	<.001	5129	4786	0.96 (0.88 to 1.05)	.40
Spinal fusion	3859	1592	0.42 (0.25 to 0.59)	<.001	7439	7473	1.02 (0.93 to 1.12)	.65
Laminectomy	3199	1512	0.51 (0.34 to 0.68)	<.001	6068	5734	1.01 (0.94 to 1.09)	.71
Coronary artery bypass graft	3099	1624	0.61 (0.45 to 0.76)	<.001	5186	4399	0.99 (0.85 to 1.12)	.83
Appendectomy	10 581	7304	0.71 (0.64 to 0.78)	<.001	18 488	17 198	0.96 (0.90 to 1.02)	.22
Lower extremity amputation	1642	1426	0.90 (0.77 to 1.03)	.16	2660	2863	1.12 (0.98 to 1.25)	.08
Cesarean delivery	32 345	30 398	0.98 (0.94 to 1.03)	.42	61 447	56 131	0.95 (0.90 to 1.01)	.11

Abbreviation: IRR, incidence rate ratio.

^a^ Weeks 12 through 18, 2020 (March 15-May 2, 2020).

^b^ Weeks 44, 2020, through 4, 2021 (October 25, 2020-January 30, 2021).

### Correlation With Weekly Rates of Patients With COVID-19

**Figure 3.**
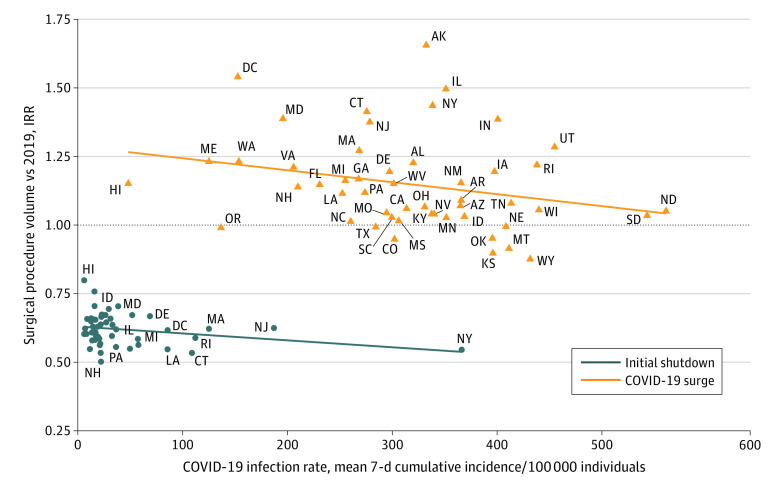
Change in Surgical Procedure Rate by Mean 7-Day Cumulative Incidence of COVID-19 per Population IRR indicates incidence rate ratio showing change in procedure volume from 2019 to 2020, estimated from Poisson regression by comparing total procedure counts during epidemiological weeks in 2020 with the corresponding weeks in 2019. Mean 7-day cumulative incidence of patients with COVID-19 per 100 000 population members by state was taken from the Centers for Disease Control and Prevention Data Tracker. A mean 7-day cumulative incidence rate was calculated for each epidemiological week and then the mean found over the initial shutdown period (ie, weeks 12-18 in 2020) and COVID-19 surge (ie, weeks 44 in 2020 through 4 in 2021). Correlation lines are plotted along the same x- and y-axis. During the initial shutdown (blue line), decrease in surgical procedure volume (by IRR) in each state was correlated with 7-day cumulative incidence rate of patients with COVID-19 (*r* = −0.00025; 95% CI, −0.0042 to −0.0009; *P* = .003). During the COVID-19 surge (orange line), there was no correlation.

During the initial shutdown period, COVID-19 incidence rate was correlated with the decrease in surgical procedure volume (as a percentage of 2019 volume) in each state (*r* = −0.00025; 95% CI, −0.0042 to −0.0009; *P* = .003) (Figure 3). State volumes of patients with COVID-19 were correlated with fewer surgical procedures during the initial shutdown (*r* = −0.00025; 95% CI −0.0042 to −0.0009; *P* = .003). During this time, the US national 7-day cumulative incidence rate of individuals with COVID-19 per 100 000 population members peaked at 66 individuals, but this does not reflect the incidence rate in the most affected state (New York, with 750 individuals with COVID-19 per 100 000 population members).^14^ In the COVID-19 surge period, when there was an 8-fold increase in the maximum national rate of COVID-19 infection (from 66 per 100 000 individuals to 532 per 100 000 individuals), the trend was similar but not statistically significant (*r* = −0.00034; 95% CI −0.00075 to 0.00007; *P* = .11). During this time, the most affected state again had a higher peak than the national incidence of infection (North Dakota, with 1388 per 100 000 individuals). During the COVID-19 surge, most states maintained surgical procedures at or above the 2019 rate (Figure 3).

## Discussion

The COVID-19 pandemic provided the opportunity to observe how hospitals limited surgical capacity quickly and effectively in preparation for a surge in volume of patients with COVID-19 during the initial pandemic response. This cohort study found that the overall rate of surgical procedures decreased by 48.0% during the initial shutdown of elective procedures compared with the same period in 2019, with the steepest decrease among ENT and musculoskeletal procedures. Later in the pandemic, when there were no federal and few state guidelines limiting elective surgical treatment, procedure rates rebounded for almost every major category of surgical procedure, for an overall procedure rate 10% lower than the 2019 baseline rate. In some categories, surgical procedure rates increased relative to the prior year during the fall and winter COVID-19 surge. There was an inverse correlation between the decrease in surgical procedures and COVID-19 disease burden at the state level during the initial shutdown but not during the COVID-19 surge. Surgical procedure volume was maintained at or above 2019 levels in most states, even those with the highest COVID incidence rates during the COIVD-19 surge.

Overall, there were approximately 670 000 fewer surgical procedures in 2020 than 2019, representing a 10% decrease. Whether these missing operations were partly associated with the 550 000 to 660 000 pandemic-related deaths^16^; decisions to defer or forgo care for nonurgent conditions, such as inguinal hernia or rotator cuff tear; or successful nonoperative management of conditions potentially requiring surgical treatment, such as appendicitis and diverticulitis, is unknown and could be a fruitful area of future research.

Our results suggest that the decrease in procedures during the initial shutdown was primarily associated with compliance with directives to curtail elective surgical procedures and perform only urgent or emergent procedures. These recommendations for stopping elective procedures were in the context of widespread uncertainty regarding disease management, transmission risks, PPE availability, inadequate testing resources, and disaster planning to prioritize access to ICU beds and ventilators. Our data suggest that the various directives from CMS, state government, and professional societies were not associated with changes in the management of health conditions that required emergency surgical procedures (eg, amputation, transplantation, and cesarean delivery). Similar to our findings, a prior analysis of nationwide claims data^17^ found that elective cataract procedures decreased by 91% and elective musculoskeletal operations by 64% in April 2020. Importantly, procedures that could be elective or urgent or emergent depending on the patient’s presenting symptoms (eg, spine, hernia, or thyroid disease) had decreased IRRs compared with such procedures in 2019, but the decrease was not to the same level as for procedures that are nearly always elective (eg, cataracts and arthroplasty).

Physician and health systems rapidly created local guidelines to manage and prioritize surgical procedures during the initial shutdown. The rate of cancer procedures, generally considered a priority, decreased as patients received alternative treatments (eg, targeted therapies, radiation, and neoadjuvant chemotherapy) or procedures for lower-risk cancers (eg, prostate or stage 0 breast cancer) were postponed.^18,19^ Patient health behaviors, such as willingness to present to an emergency department, may have been associated with a fear of COVID-19 transmission. At 5 institutions across the US, for example, the volume of patients with uncomplicated appendicitis decreased after declaration of the pandemic.^20^ The decrease in rates of surgical procedures over the 7-week initial shutdown was almost certainly multifactorial, associated with hospital policies, patient behavior, and physician clinical judgement.

Compared with the initial pandemic response, in March through April 2020, there are limited data to fully explain the rapid and sustained rebound of most surgical procedure rates during the COVID-19 surge in the fall and winter of 2020, when the volume of patients with COVID-19 throughout the US increased 8-fold. During the COVID-19 surge, surgical procedure volume was determined by individual hospitals and systems rather than national or local policy. In this period, there was no correlation of surgical IRR with COVID-19 disease burden. Our findings suggest that in the absence of national recommendations and state government policies, increased rates of patients with COVID-19 were likely not the strongest factor associated with surgical procedure volume. Rather, these findings suggest that health systems’ surgical services responded effectively and hospitals adapted elective surgical procedure policies based on local needs and resources. Indeed, we observed a rebound to prepandemic levels for every major surgical procedure category except ENT procedures. It is plausible that hospitals learned how to manage risks during the initial shutdown and used that new knowledge to balance the medical and financial obligation to provide surgical care and reduce backlogged patients,^21,22,23^ limit COVID-19 transmission, and preserve hospital resources for surging populations of patients with COVID-19. Additionally, by the time of the fall and winter surge, hospitals had critical COVID-19 testing capacity and the recognition that ambulatory surgical procedures could continue without compromising hospital bed capacity. We note that US in-hospital mortality for patients testing positive for COVID-19 peaked in April 2020 (19.7%) and decreased in all age groups by 50% by June 2020.^24^ Infection control procedures were associated with the near disappearance of nosocomial transmission and infections among health care workers.^24,25^ Financial factors were also likely associated with restoration of surgical procedure volume quickly, but an economic analysis was beyond the scope of this investigation, as was characterization of clinician and patient risk aversion or acceptance. Of note, ENT procedures by nature place the surgeon in closest contact with the patient airway and secretions and represented the one category of procedures that did not return to 2019 levels. Vaccine availability for health care workers was established at the end of this study period and was likely associated with many physicians feeling safer performing procedures.

### Limitations

This study is subject to several limitations that must be noted. First, our data are limited to patients with insurance that uses Change Healthcare for claims processing. However, the large sample size and rapidity of data collection suggest that this data set was highly representative at the national level. Second, we did not include data on diagnostics, race, or other social determinants of health in this analysis and cannot make claims about the association of underlying conditions with surgical treatment decisions or potential disparities in operative access. The CPT codes used in this analysis were based on expert discretion about what would reasonably be performed in an operating room. However, to maintain consistency with prior research, we based our clinical categories on the Healthcare Cost and Utilization Project. Given that our analysis included only the first surgical procedure claim per patient per calendar day, we did not capture the rare events of operative procedures performed on different body systems within the same day. To preserve patient privacy, data were analyzed at the state level and therefore cannot reveal trends within states. Future research should examine potential disparate experiences and outcomes among different hospitals settings and patient populations.

## Conclusions

This study found a 48.0% decrease in total surgical procedures during the 7 weeks after the declaration of the COVID-19 pandemic and a rapid return to baseline or even greater operation rates for nearly all surgical procedure categories. Patient flow through operating rooms was maintained even during the highest per capita rates of patients with COVID-19 in the fall and winter of 2020 to 2021. Hospitals and surgical centers recovered quickly after the initial shutdown, suggesting that adaptability, resiliency, increased knowledge of limiting transmission, and financial factors may have played a role in reestablishment of baseline surgical procedure volumes even in the setting of substantially increased COVID-19 disease burden. Our findings and future work focused on procedure types at a more granular level may be used to inform disaster planning, with the goal of limiting health care shutdowns and optimizing the maintenance of surgical procedure capacity during public health crises.

## Notes

Supplement. **eTable 1.** Surgical Procedure Volume and Incidence Ratio Rate During Initial Shutdown and COVID-19 Surge vs Prepandemic Rate **eTable 2.** Surgical Procedure Volume by Subcategory During Initial Shutdown and COVID-19 Surge vs Prepandemic Rate **eFigure.** Rates of Exemplar Procedures During Initial Shutdown and COVID-19 Surge Compared With Prepandemic Rate
