# Supplementary material for: Trends in US Surgical Procedures and Health Care System Response to Policies Curtailing Elective Surgical Operations During the COVID-19 Pandemic
Source: JAMA Netw Open. 2021 Dec 8;4(12):e2138038. doi: 10.1001/jamanetworkopen.2021.38038 (PMC8655602; doi:10.1001/jamanetworkopen.2021.38038)
Supplement: Supplement. — eTable 1. Surgical Procedure Volume and Incidence Ratio Rate During Initial Shutdown and COVID-19 Surge vs Prepandemic Rate eTable 2. Surgical Procedure Volume by Subcategory During Initial Shutdown and COVID-19 Surge vs Prepandemic Rate eFigure. Rates of Exemplar Procedures During Initial Shutdown and COVID-19 Surge Compared With Prepandemic Rate [file jamanetwopen-e2138038-s001.pdf]

Supplemental Online Content

Mattingly AS, Rose L, Eddington HS, et al. Trends in US surgical procedures and health care system response to policies curtailing elective surgical operations during the COVID-19 pandemic. *JAMA Netw Open*. 2021;4(12):e2138038. doi:10.1001/jamanetworkopen.2021.38038

eTable 1. Surgical Procedure Volume and Incidence Ratio Rate During Initial Shutdown and COVID-19 Surge vs Prepandemic Rate
eTable 2. Surgical Procedure Volume by Subcategory During Initial Shutdown and COVID-19 Surge vs Prepandemic Rate
eFigure. Rates of Exemplar Procedures During Initial Shutdown and COVID-19 Surge Compared With Prepandemic Rate

This supplemental material has been provided by the authors to give readers additional information about their work.

© 2021 Mattingly AS et al. *JAMA Network Open.*

eTable 1. Surgical Procedure Volume and Incidence Ratio Rate During Initial Shutdown and COVID-19 Surge vs Prepandemic Rate

Surgical Category	Initial Shutdown Weeks 12-18, 2020[a]				COVID-19 Surge Week 44, 2020- Week 4, 2021[b]			
	Volume[c]		IRR (95% CI)[d]	P value	Volume[c]		IRR (95% CI)[d]	P value
	2019	2020			2019	2020		
**Overall**	905,444	458,469	0.52 (0.44-0.60)	<0.001	797,510	756,377	0.97 (0.95-1.00)	0.10
Cataract	13,564	1,396	0.11 (-0.11-0.32)	0.03	24,430	23,797	1.00 (0.90-1.09)	0.95
ENT[e]	36,702	10,945	0.30 (0.13-0.46)	<0.001	60,090	41,701	0.70 (0.65-0.75)	<0.001
Musculoskeletal	150,145	53,473	0.36 (0.21-0.52)	<0.001	293,100	279,679	0.97 (0.89-1.05)	0.51
Nervous	43,054	16,829	0.41 (0.26-0.55)	<0.001	82,361	80,291	1.01 (0.95-1.07)	0.71
General	128,594	57,318	0.46 (0.33-0.59)	<0.001	237,452	222,696	0.97 (0.92-1.02)	0.22
Urology	33,069	15,649	0.48 (0.36-0.61)	<0.001	60,846	60,708	1.02 (0.94-1.09)	0.63
Cardiovascular	42,246	21,967	0.55 (0.44-0.65)	<0.001	73,873	69,310	0.98 (0.93-1.04)	0.61
OB-GYN	92,934	53,205	0.60 (0.49-0.70)	<0.001	174,908	165,044	0.98 (0.94-1.03)	0.42
Skin	358,959	223,975	0.63 (0.55-0.71)	<0.001	635,449	628,399	1.00 (0.95-1.04)	0.87
Thoracic	5,633	3,314	0.64 (0.53-0.76)	<0.001	9,829	9,321	1.04 (0.94-1.13)	0.46
Transplant	544	398	0.79 (0.59-1.00)	0.08	1,021	986	1.05 (0.82-1.27)	0.68

[a] March 15, 2020- May 2, 2020
[b] October 25, 2020- January 30, 2021
[c] Absolute number of surgical procedures
[d] Incidence rate ratio (95% confidence interval), estimated from Poisson regression by comparing total procedure counts during epidemiological weeks in 2020 to the corresponding weeks in 2019
[e] Ears, Nose, Throat

© 2021 Mattingly AS et al. *JAMA Network Open.*

eTable 2. Surgical Procedure Volume by Subcategory During Initial Shutdown and COVID-19 Surge vs Prepandemic Rate

**Surgical Category**	**Initial Shutdown** Weeks 12-18, 2020[a]				**COVID-19 Surge** Week 44, 2020- Week 4, 2021[b]			
	Volume[c]		IRR (95% CI)[d]	P value	Volume[c]		IRR (95% CI)[d]	P value
	2019	2020			2019	2020		
Cataract	13,564	1,396	0.11 (-0.11-0.32)	0.03	24,430	23,797	1.00 (0.90-1.09)	0.95
Arthroplasty	53,328	9,737	0.18 (-0.01-0.37)	0.001	107,235	98,993	0.90 (0.79-1.01)	0.11
Abdominal Hernia Repair	27,014	6,178	0.23 (0.08-0.39)	<0.001	52,330	46,484	0.91 (0.83-0.98)	0.02
Male Reproductive	6,103	1,577	0.26 (0.12-0.39)	<0.001	11,610	12,133	1.04 (0.94-1.14)	0.43
Non-fracture Musculoskeletal	65,297	18,945	0.30 (0.13-0.46)	<0.001	127,448	121,899	0.98 (0.91-1.06)	0.62
Ear, Nose, Throat	36,702	10,945	0.30 (0.13-0.46)	<0.001	60,090	41,701	0.70 (0.65-0.75)	<0.001
Gynecology	54,219	18,276	0.35 (0.19-0.51)	<0.001	102,267	98,220	0.99 (0.93-1.05)	0.80
Skull, Brain, Meninges, and Nerves	20,824	7,401	0.37 (0.22-0.52)	<0.001	40,130	39,413	1.02 (0.96-1.09)	0.50
Spine	22,230	9,428	0.44 (0.30-0.58)	<0.001	42,231	40,878	1.00 (0.93-1.07)	0.97
Prostate	5,617	2,563	0.48 (0.33-0.63)	<0.001	10,006	9,692	1.02 (0.91-1.13)	0.75
Grafts, Flaps and other Reconstruction	30,649	15,549	0.52 (0.40-0.63)	<0.001	56,690	56,525	1.01 (0.94-1.08)	0.72
Solid Organ: HPB[e], Spleen	25,549	12,369	0.52 (0.37-0.66)	<0.001	46,207	42,910	1.00 (0.94-1.05)	0.90
Hollow Viscus, Peritoneal Cavity	61,689	31,484	0.52 (0.42-0.62)	<0.001	111,703	106,839	0.98 (0.93-1.02)	0.37
Breast, Endocrine	14,342	7,287	0.52 (0.36-0.69)	<0.001	27,212	26,463	1.00 (0.93-1.08)	0.94
Vascular	29,885	15,315	0.55 (0.45-0.64)	<0.001	51,873	49,180	1.01 (0.95-1.07)	0.78
Cardiac	12,361	6,652	0.55 (0.44-0.66)	<0.001	22,000	20,130	0.93 (0.86-1.00)	0.04
Kidney, Bladder, Urethra	21,349	11,509	0.55 (0.43-0.67)	<0.001	39,230	38,883	1.01 (0.94-1.08)	0.78
Excision of Skin and Soft Tissue	214,614	118,204	0.58 (0.49-0.68)	<0.001	369,927	346,410	0.99 (0.94-1.04)	0.78
Thoracic	5,633	3,314	0.64 (0.53-0.76)	<0.001	9,829	9,321	1.04 (0.94-1.13)	0.46
Incision, Drainage, and Debridement	113,696	90,222	0.73 (0.68-0.78)	<0.001	208,832	225,464	1.00 (0.94-1.05)	0.89
Abortion	6,370	4,531	0.77 (0.67-0.87)	<0.001	11,194	10,693	1.03 (0.95-1.12)	0.45
Transplant	544	398	0.79 (0.59-1.00)	0.08	1,021	986	1.05 (0.82-1.27)	0.68
Amputation	6,091	4,904	0.81 (0.69-0.92)	0.004	10,832	10,572	0.98 (0.90-1.06)	0.58
Fracture	25,429	19,887	0.86 (0.78-0.94)	0.001	47,585	48,215	1.11 (1.04-1.19)	0.002

© 2021 Mattingly AS et al. *JAMA Network Open.*

Cesarean Section	32,345	30,398	0.98 (0.94-1.03)	0.42	61,447	56,131	0.95 (0.90-1.01)	0.11

[a] March 15, 2020- May 2, 2020
[b] October 25, 2020- January 30, 2021
[c] Absolute number of surgical procedures
[d] Incidence rate ratio (95% confidence interval), estimated from Poisson regression by comparing total procedure counts during epidemiological weeks in 2020 to the corresponding weeks in 2019
[e]HPB= hepato-pancreato-biliary

© 2021 Mattingly AS et al. *JAMA Network Open.*

eFigure. Rates of Exemplar Procedures During Initial Shutdown and COVID-19 Surge Compared With Prepandemic Rate

eFigure 1 Legend: Initial Shutdown = March 15, 2020- May 2, 2020; COVID-19 Surge = October 25, 2020- January 30, 2021; IRR= Incidence rate ratio, estimated from Poisson regression by comparing total procedure counts during epidemiological weeks in 2020 to the corresponding weeks in 2019. Error bars indicate 95% confidence interval. Exemplar procedures followed a predicted decline in volume from 2019 during the Initial Shutdown, based on level of urgency. Groin hernia repair and knee arthroplasty remained slightly lower than 2019 levels during the weeks representing the COVID-19 Surge. See Table 2 for exact values.

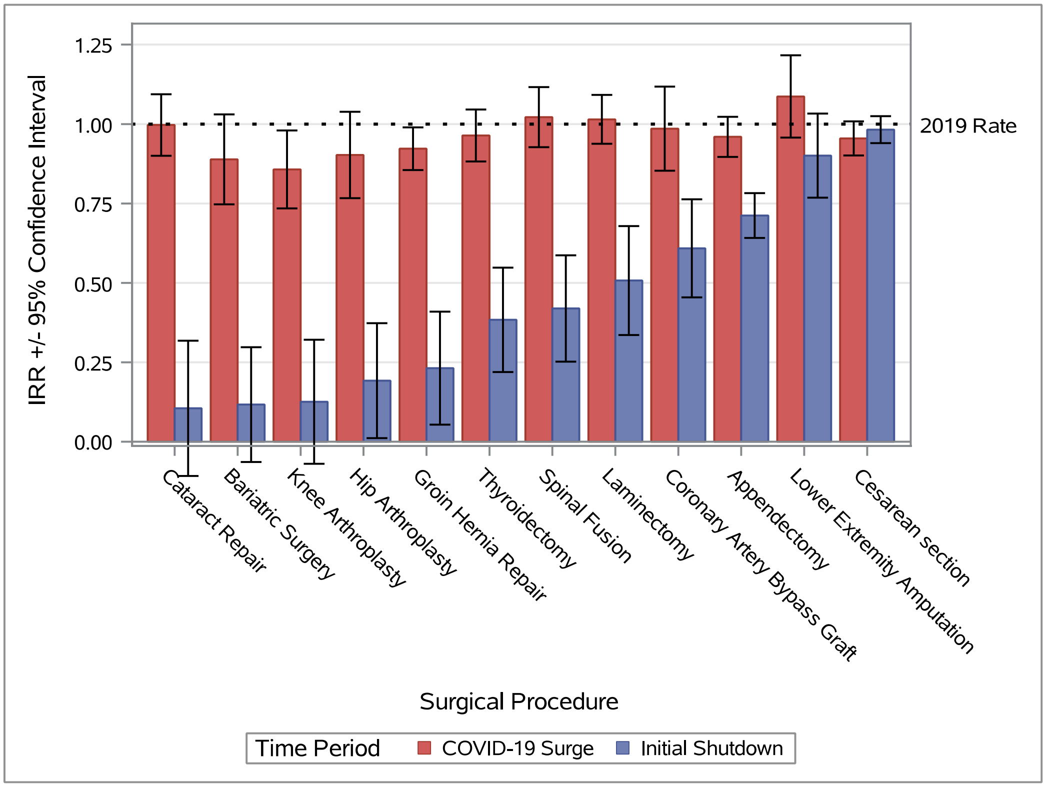

© 2021 Mattingly AS et al. *JAMA Network Open.*
